# A Trend for Increased Risk of Revision Surgery due to Deep Infection following Fast-Track Hip Arthroplasty

**DOI:** 10.1155/2016/7901953

**Published:** 2016-02-29

**Authors:** Einar Amlie, Anners Lerdal, Caryl L. Gay, Øystein Høvik, Lars Nordsletten, Sigbjørn Dimmen

**Affiliations:** ^1^Orthopedic Research Group, Lovisenberg Diakonale Hospital, Nydalen, P.O. Box 4970, 0440 Oslo, Norway; ^2^Department of Nursing Science, Institute of Health and Society, Faculty of Medicine, University of Oslo, Blindern, P.O. Box 1130, 0450 Oslo, Norway; ^3^Department of Family Health Care Nursing, University of California, San Francisco, Box 0606, San Francisco, CA 94143, USA; ^4^Institute of Clinical Medicine, Faculty of Medicine, University of Oslo, Nydalen, P.O. Box 4956, 0450 Oslo, Norway; ^5^Oslo University Hospital, University of Oslo, 0450 Oslo, Norway

## Abstract

Rates of revision surgery due to deep infection following total hip arthroplasty (THA) increased at a Norwegian hospital following implementation of fast-track procedures. The purpose of this study was to determine whether selected demographic (age and sex) and clinical (body mass index, American Society of Anesthesiologists (ASA) classification, surgery duration, length of hospital stay, cemented versus uncemented prosthesis, and fast-track procedures) factors were associated with higher risk of revision surgery due to deep infection following THA. In a prospective designed study 4,406 patients undergoing primary THA between January 2001 and January 2013 where included. Rates of infection-related revision surgery within 3 months of THA were higher among males and among patients who received fast-track THA. Adjusting for sex and age, the implemented fast-track elements were significantly associated with increased risk of revision surgery. Risk of infection-related revision surgery was unrelated to body mass index, physical status, surgery duration, length of hospital stay, and prosthesis type. Because local infiltration analgesia, drain cessation, and early mobilization were introduced in combination, it could not be determined which component or combination of components imposed the increased risk. The findings in this small sample raise concern about fast-track THA but require replication in other samples.

## 1. Introduction

Fast-track (also called enhanced recovery and accelerated track) total hip arthroplasty (THA) has reduced the length of stay (LOS) in hospital from 4–10 days to 2–4 days [1–3]. The aim of fast-track surgery is to enhance functional recovery and reduce perioperative morbidity and hospitalization by combining optimal clinical care with organizational factors [4]. A Danish study [3] showed that pain was one of the most important clinical factors associated with longer stay in hospital. Thus, multimodal analgesic treatment has been a cornerstone of the fast-track treatment strategy. Pain treatment includes opioid-sparing regimens and may include use of local infiltration analgesia (LIA) [5, 6]. Studies have shown that patients with LIA require less narcotic medication, have reduced LOS in hospital, and are able to walk sooner than patients with epidural analgesia [7, 8]. However, high-volume LIA was shown in a randomized controlled trial to have no additional benefit for patients treated with multimodal opioid-sparing analgesia for fast-track bilateral hip surgery [5, 9]. Type of anesthesia and management of symptoms, particularly pain, have important roles in postoperative recovery and are thus critical for fast-track arthroplasty [10]. Furthermore, early mobilization (i.e., within a few hours postoperatively) has been an important contributor to accelerated recovery [11, 12] as has been the optimization of hospital logistics, such as timely access to required radiography and physiotherapy [3].

Lovisenberg Diakonale Hospital implemented elements of fast-track hip arthroplasty in 2009. During the implementation period, rates of postoperative deep infection necessitating revision surgery increased, which led to revision of the treatment protocol. Thus, the aim of this study was to determine whether demographic (age and sex) or clinical (body mass index (BMI), American Society of Anesthesiologists (ASA) classification, LOS in hospital, surgery duration, prosthesis type (cemented or uncemented), and fast-track procedures) factors were related to risk of infection-related revision surgery in patients undergoing THA.

## 2. Materials and Methods

Early complications after prosthesis surgery have been registered prospectively since 2001. Medical records were reviewed for all patients who met the inclusion criteria, that is, had primary THA with a posterolateral approach, using a cemented or uncemented prosthesis, between January 2001 and January 2013. Patients with partially implemented fast-track procedures after termination of LIA and drain cessation were excluded (i.e., surgeries that included early mobilization and standardized pain treatment regimen only, *n* = 133). The primary outcome variable was whether the patient had revision surgery due to deep infection within three months of the initial surgery. Deep infection was determined by clinical judgment along with factors such as prolonged drainage and/or increasing infection parameters (erythrocyte sedimentation rate (ESR) and c-reactive protein (CRP)). Revision surgeries included irrigation and debridement and exchange of modular components. Data collected from the medical record included patient age and sex, BMI, ASA classification, surgery duration, and LOS in hospital. The dataset was then anonymized and exported from the quality register into a separate database.

The fast-track THA protocol was initiated in August 2009 and included four main components: (a) LIA consisting of 200 mg ropivacaine (100 mL) combined with 2 mg adrenaline (2 mL) for a total solution of 102 mL injected into the capsule and surrounding muscles and 100 mg ropivacaine (50 mL) without adrenaline injected subcutaneously after closure of the fascia; (b) cessation of negative vacuum suction drain, which, prior to fast-track implementation, had been part of the standard surgical protocol; (c) early mobilization, which involved mobilizing the patient to bedside upright position the evening of the surgery rather than the next day; and (d) a standardized pre- and postoperative pain management regimen, which involved gabapentin 300 mg ×2, paracetamol 1 g ×4, and celecoxib 200 mg ×2.

**Figure 1:**
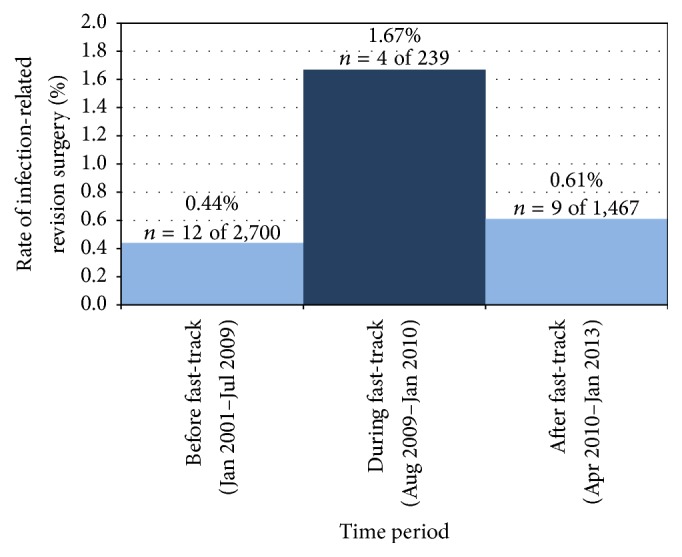
Rates of revision surgery due to deep infection following primary THA based on continuous quality surveillance data. The 6-month period during which the full fast-track protocol was implemented had a significantly higher rate of revision surgery due to deep infection within 3 months of the primary THA surgery. Infection-related revision surgery rates during the periods before and after the fast-track protocol was implemented were not significantly different. Surgeries including only partial implementation of the fast-track procedures (performed Jan to Apr 2010) were excluded from the analysis.

Infection rates were monitored over time as part of the hospital's ongoing quality assurance efforts. Due to a noticeable increase in infection rates (Figure 1) and infection-related revision surgeries, components of the fast-track protocol were discontinued in a step-wise fashion in an effort to reduce infections. LIA was discontinued and suction drain resumed in Jan 2010, and mobilization was delayed until the day after surgery starting in Apr 2010. Gabapentin was discontinued in Nov 2011, but because this represented only a partial change to the standardized pain management regimen, it was not specifically addressed in this analysis. Antibiotic regimen included a dose of cefalotin 2 g ×4 (or clindamycin 600 mg ×3 if allergic to cefalotin) given within 30 minutes preoperatively and the regimen was unchanged during the course of the study. Medical records were reviewed to determine which fast-track components were included in each surgery performed during the study period. Patients were then categorized into two groups: those who had surgeries that included the four main components of the fast-track protocol (generally performed between August 2009 and January 2010) and those who had standard THA surgeries (performed before August 2009 or after April 2010). Patients who received only the standardized pain treatment regimen (administered between April 2010 and November 2011) were included in the standard THA surgery group. Prosthesis type was categorized as cemented or uncemented based on the surgical procedure codes in the medical record. Cemented prostheses with gentamicin (Spectron-EF® and Reflection® Cemented All Polyethylene, Smith & Nephew Orthopaedics, Memphis, TN, USA) were the primary implant used until November 2009, after which uncemented prosthesis implants (Reflection® Acetabular Cup System, Smith & Nephew Orthopaedics, Memphis, TN, USA; Corail® Total Hip System, DePuy Orthopaedics, Warsaw, IN, USA) were used exclusively. A posterolateral approach to the joint was routinely used in THA for the duration of the study period.

### 2.1. Statistical Analysis

Descriptive statistics were used to summarize demographic and clinical characteristics. Group comparisons on continuous variables were conducted using independent sample Student's *t*-tests. Associations between variables were assessed using chi-square test of independence for categorical variables, or when expected cell frequencies were low (<5), Fisher's Exact Test was used instead. Effect sizes were also reported; Cohen's *d* values > 0.40 and phi values > 0.20 were considered clinically meaningful [13].

Logistic regression analysis was used to evaluate factors associated with infection-related revision surgery while controlling for other relevant variables. Odds ratios (ORs) were used to quantify the unique contribution of each predictor to revision surgery risk and an OR ≥ 2.0 was considered clinically meaningful. ORs yield acceptable approximations or relative risk when the outcome of interest is rare, as is the case with infection-related revision surgery [14]. Logistic regression analyses adjusted for both age and sex, given their prior association with postoperative infection, and any additional variables in Table 1 that were associated with infection-related revision surgery at a significance level of *p* < 0.20 were also included as covariates. The significance level for all other analyses was set at *p* < 0.05. All analyses were conducted using SPSS Version 20 (SPSS, Inc., Somers, NY, USA).

### 2.2. Ethics

This quality assurance study was performed as part of continuous ongoing quality surveillance in the hospital's orthopaedic department and was approved by the hospital ethics committee.

## 3. Results

The total sample consisted of the 4,406 patients who met the inclusion criteria. Demographic and clinical characteristics for the 4,406 patients included in the analysis are summarized in Table 1. The majority (72%) of the sample was female, and although there were statistically significant sex differences with respect to both demographic and clinical characteristics, the effect sizes were small and none met the criterion for clinical significance.

### 3.1. Factors Associated with Infection-Related Revision Surgery

All variables in Table 1 were evaluated for their association with infection-related revision surgery within three months of the initial surgery. Rates of revision surgery due to deep infection were significantly higher among men than women and among patients who had fast-track THA compared with those who had standard THA (Table 2). Revision surgery due to deep infection was unrelated to patient age, BMI, ASA classification, surgery duration, and LOS in hospital. Because uncemented prostheses were more common with fast-track surgery than standard surgery (70% versus 45%, *χ* ^2^ = 56.7, *p* < 0.001), the relationship between prosthesis type and risk of revision surgery due to deep infection was evaluated separately for each surgical subgroup. Although all four infection-related revision surgeries associated with fast-track surgery occurred in patients with an uncemented prosthesis, the relationship between prosthesis type and risk of infection-related revision surgery was not significant among either fast-track or standard THA surgeries. In the multiple regression analysis (Table 3), fast-track THA was significantly predictive of revision surgery due to deep infection (*p* = 0.030), even when controlling for the potentially confounding effects of male gender and older age.

### 3.2. Rates of Infection-Related Revision Surgery before, during, and after Fast-Track Implementation

The rate of infection-related revision surgery before fast-track surgery was implemented was 0.44% (*n* = 12 of 2,700), which increased to 1.67% (*n* = 4 of 239) during the fast-track period, and returned to a rate of 0.61% (*n* = 9 of 1,467) once the fast-track procedures were discontinued (Figure 1). Patients undergoing primary THA according to the fast-track protocol had an adjusted risk of revision surgery due to deep infection during the first 3 postoperative months that was more than three times higher (OR = 3.3, 95% CI 1.125–9.772, *p* = 0.03) than patients who had standard THA (Table 3).

These rates indicate a statistically significant increase from the period before fast-track to the period when fast-track was fully implemented (*χ* ^2^ = 6.13, *p* = 0.013). Although the subsequent decrease in infection rate following the discontinuation of fast-track did not reach statistical significance (*χ* ^2^ = 3.05, *p* = 0.081), the infection rate after fast-track was discontinued did not differ from the infection rate before it was implemented (*χ* ^2^ = 0.54, *p* = 0.462), suggesting that the infection rate had returned to the earlier level.

## 4. Discussion

In this study, patients undergoing primary THA according to the fast-track protocol had more than three times the risk of developing a deep infection that necessitated revision surgery during the first 3 postoperative months compared to patients who had standard THA. Of the clinical factors evaluated in this study, fast-track surgery was the only one with a significant relationship to increased risk for infection-related revision surgery after controlling for male gender and older age. Prior studies indicate an association between increased infection risk and older age and male sex [15–18], but only sex was associated with infection-related revision surgery in our study. To our knowledge, this is the first study to report a clinically relevant relationship between fast-track THA surgery and increased risk for postoperative deep infection necessitating revision surgery, adjusting for other infection risk factors.

A retrospective study of 1,180 patients from the Netherlands [19] reported no statistically significant differences in complications after introduction of fast-track THA surgery. Although a nonsignificant decrease in postoperative infection rates (from 3.2% to 0.9%) was observed following initial implementation of the fast-track procedures in that study, there also seemed to be a slight increase in infections (from 0.9% to 1.8%) between partial and full implementation of the procedures. However, given the low base rate of infections following THA, the sample size in that study was likely too small to detect such subtle changes in infection rates. A basic power analysis indicates that a sample size of more than 5,000 patients would be needed to detect a 100% increase in infection rates that low, and even more if the groups were not of equal size. Because deep infection rates following THA are generally quite low, large samples are needed in order to identify risk factors, particularly those with more subtle effects. It is therefore possible that the absence of an association between fast-track THA and infection risk in prior studies is due to the fact that smaller samples lack sufficient statistical power for identifying such associations.

A prospective study of infection after primary THA based on data from the Norwegian Arthroplasty Register during the years 2005–2009 reported an incidence of infection of 3% during the first year after surgery [17]. Their rate of THA revision surgery due to infection (0.7%) is comparable to that observed in the current study. In addition, a similar Nordic study of 432,168 patients undergoing THA between 1995 and 2009 reported an overall infection rate 0.6%, as well as a significant increase in infection rates requiring revision from 0.46% to 0.71% over the course of the study [20]. Their study did not evaluate fast-track procedures. However, fast-track surgeries have become more common in recent years, and this might partly explain the slight increase in infection rates. In contrast, a study of more than 1.4 million patients undergoing primary THA in the United States reported a trend of decreasing rates of postoperative infections [21].

Because the fast-track procedures were evaluated in combination, it is not clear from this analysis which component or combination of components confers the increased risk of infection-related revision surgery. Moreover, alternative explanations for the increased rate of revisions due to deep infections need to be considered. Adrenaline was added to the ropivacaine solution under sterile conditions in the operating theater with laminar air flow (LAF) rather than having the solution prepared by the pharmacy. Although it cannot be ruled out that the solution was contaminated during preparation, we have no reason to believe that this was the case and infections occurred across a number of different surgical teams. Increased risk of deep infection has been reported with the use of cyclooxygenase- (cox-) 2 inhibitors in patients undergoing coronary artery bypass grafting surgery [22]. In the present study, infection risk decreased after the other fast-track procedures were discontinued, despite continued use of cox-2 inhibitors. However, this decrease was not significant and the subsequent rate of infection-related revision surgery did not reach the low level observed before fast-track procedures were introduced. Thus, it is possible that use of cox-2 inhibitors was associated with a slightly higher infection risk, which may have been amplified when combined with other fast-track procedures. Uncemented prostheses (without any antibiotic as in the cemented prostheses) were also used routinely after the fast-track period, and further studies are needed to determine the effects of cox-2 inhibitors and uncemented prostheses on postoperative deep infections after THA.

One possible mechanism by which the fast-track procedures could have resulted in increased infections requiring revision surgery is that high-volume local anesthetics combined with the absence of vacuum suction drain might increase pressure in the tissue and affect circulation or increase oozing from the wound.

Given the notable benefits of fast-track THA procedures, including shorter LOS in hospital and faster recovery, the increased risk of infection-related revision surgery described in this study may be acceptable for some patients, particularly those with few additional infection risk factors. However, for patients already at high risk for infection due to comorbidities, advanced age, and male sex, further exacerbation of their risk of infection-related revision surgery may require additional consideration.

This study has several strengths and limitations that need to be considered. The primary strengths of the current study are its prospective design and large sample, which allowed for the evaluation of various infection risk factors in a setting with generally low rates of deep infection following primary THA. Other strengths included the lack of variation in surgical approach (all were posterolateral) and accounting for multiple risk factors, so that the unique contribution of each could be determined after controlling for the effects of the others. However, the findings also need to be considered in light of several study limitations. The most notable limitation is that, given the quasiexperimental nature of the study design, it cannot be ruled out that the observed increase and subsequent decrease in deep infections following THA were due to factors other than the fast-track procedures, such as the learning curve for the new surgical protocol or the switch to uncemented prostheses. The findings could also be due to other potential risk factors not included in this analysis, although none of the patients with infection had other known risk factors, such as diabetes, smoking, obesity, rheumatoid arthritis, or malignant disease. A randomized controlled trial would be a stronger study design, but, given the low incidence of deep infections following primary THA, a very large sample would be required. In the current study, the number of surgeries performed with LIA was relatively small, as was the number of infection-related revision surgeries in the LIA group. Although the findings were statistically significant, definitive conclusions should not be based on such small numbers. In addition, at the time of data collection (from 2001 to 2013), the evaluation of deep infection and need for revision surgery was based on clinical judgment, and the latest criteria for periprosthetic joint infection [23] were not published until 2011. Furthermore, data were retrieved from a large set of medical records, which might have increased the risk for errors. However, variables included in the study have been a part of the quality assurance system with 98% complete data and a daily reliability assessment by trained personnel. Given these limitations, the current findings need to be replicated in other samples before definitive conclusions can be drawn.

Several additional points affecting the generalizability of these findings are also worth noting. First, this study focused on deep infections that warranted revision surgery, and less serious infections were not evaluated [24]. Second, the hospital at which the current study was conducted only performs elective THA surgery, and the risk factors for post-THA infection identified in this study may differ from those in departments performing emergency surgeries [25]. Lastly, since fast-track THA protocols consist of different types and combinations of interventions that can vary by hospital, our findings cannot be generalized to fast-track THA in general and need to be interpreted in light of the specific procedures used in this study. An evaluation of the clinical effects of each protocol component, alone and in combination, on patient outcomes is warranted.

In conclusion, the present study reports an increased risk of revision surgery due to deep infection following a relatively brief implementation of fast-track THA and a subsequent decrease when it was discontinued. Fast-track THA was associated with increased risk of infection-related revision surgery even after adjusting for other relevant factors. While the study's limitations prevent definitive conclusions, the current findings suggest that further research in this area is warranted. If these findings are replicated in other samples, it will be important to determine the mechanisms by which fast-track THA might contribute to increased infection risk and thereby identify potential mitigation strategies.

## Figures and Tables

**Table 1:** Patient demographic and clinical characteristics (*n* = 4,406).

Characteristic	Men *n* = 1,246	Women *n* = 3,160	Statistics and effect sizes
Age in years (*n* = 4,403)	67.4 (10.8)	70.5 (10.0)	*t* = 8.63^a^, *p* < 0.001; *d* = 0.29
Range in years	26 to 92	24 to 94	
Age group (*n* = 4,403)			*χ* ^2^ = 52.1, *p* < 0.001; phi = 0.109
≤70 years (*n* = 2,195)	728 (58.5%)	1,467 (46.4%)
>70 years (*n* = 2,208)	516 (41.5%)	1,692 (53.6%)
Body mass index (*n* = 2,831)^b^	26.9 (3.9)	25.9 (4.6)	*t* = 5.79^a^, *p* < 0.001; *d* = 0.23
Range	17.8 to 40.1	15.6 to 66.2	
ASA score (*n* = 3,724)^b^			*χ* ^2^ = 26.3, *p* < 0.001; phi = 0.084
(1) Healthy (*n* = 608)	188 (17.6%)	420 (15.9%)
(2) Mild disease (*n* = 2,543)	675 (63.0%)	1,868 (70.7%)
(3) Severe disease (*n* = 560)	207 (19.3%)	353 (13.4%)
(4) Life-threatening disease (*n* = 2)	1 (0.1%)	1 (0.0%)
Surgery duration (hrs : mins; *n* = 3,713)^b^	1 : 10 (0 : 19)	1 : 07 (0 : 17)	*t* = 3.86, *p* < 0.001; *d* = 0.13
Length of hospital stay (days)	7.28 (2.81)	7.85 (3.34)	*t* = 5.40, *p* < 0.001; *d* = 0.18
Range in days	1 to 27	1 to 95	
Surgery type			*χ* ^2^ = 1.20, *p* = 0.274; phi = 0.016
Standard THA (*n* = 4,167)	1,171 (94.0%)	2,996 (94.8%)
Fast-track THA (*n* = 239)	75 (6.0%)	164 (5.2%)
Prosthesis type			*χ* ^2^ = 40.3, *p* < 0.001; phi = 0.096
Cemented (*n* = 2,368)	575 (46.1%)	1,793 (56.7%)
Uncemented (*n* = 2,038)	671 (53.9%)	1,367 (43.3%)

Note. THA: total hip arthroplasty. While many of the gender differences are statistically significant, the effect sizes are small.

^a^Separate variance *t*-test.

^b^Body mass index was not routinely recorded prior to January 2007, and ASA classification and surgery duration were not routinely recorded prior to April 2004, and thus reduced sample sizes were available for these variables. ASA scores (3) and (4) were combined for analysis.

**Table 2:** Rates of infection-related revision surgery by patient demographic and clinical characteristics (*n* = 4,406).

Characteristic	Infection-related revision surgery within 3 months?		Statistics and effect sizes
	No *n* = 4,381 (99.43%)	Yes *n* = 25 (0.57%)	
Gender			*χ* ^2^ = 7.01, *p* = 0.008; phi = 0.040
Male (*n* = 1,246)	1,233 (98.96%)	13 (1.04%)
Female (*n* = 3,160)	3,148 (99.62%)	12 (0.38%)
Age in years (*n* = 4,406)	69.6 (10.3)	71.7 (9.6)	*t* = 1.02, *p* = 0.308; *d* = 0.21
Age group (*n* = 4,406)			*χ* ^2^ = 0.98, *p* = 0.323; phi = 0.015
≤70 years (*n* = 2,195)	2,185 (99.54%)	10 (0.46%)
>70 years (*n* = 2,208)	2,193 (99.32%)	15 (0.68%)
Body mass index (*n* = 2,831)^a^	26.2 (4.4)	25.9 (4.7)	*t* = 0.36, *p* = 0.715; *d* = 0.07
ASA score (*n* = 3,713)^a^			*χ* ^2^ = 0.67, *p* = 0.880; phi = 0.013
(1) Healthy (*n* = 608)	604 (99.34%)	4 (0.66%)	
(2) Mild disease (*n* = 2,543)	2,528 (99.41%)	15 (0.59%)	
(3) Severe disease (*n* = 560)	555 (99.11%)	5 (0.89%)	
(4) Life-threatening disease (*n* = 2)	2 (100%)	0 (0%)	
Surgery duration (hrs : mins; *n* = 3,713)^a^	1 : 08 (0 : 17)	1 : 09 (0 : 16)	*t* = 0.28, *p* = 0.780; *d* = 0.06
Length of hospital stay (days)^b^	7.66 (2.80)	7.96 (3.32)	*t* = 0.53, *p* = 0.598, *d* = 0.10
Surgery type			*χ* ^2^ = 5.48, *p* = 0.019; phi = 0.035
Standard THA (*n* = 4,167)	4,146 (99.50%)	21 (0.50%)
Fast-track THA (*n* = 239)	235 (98.33%)	4 (1.67%)
Prosthesis type (*n* = 4,178)^c^			
Standard THA (*n* = 4,167)			*χ* ^2^ = 0.06, *p* = 0.802; phi = 0.004
Cemented (*n* = 2,296)	2,285 (99.52%)	11 (0.48%)
Uncemented (*n* = 1,871)	1,861 (99.47%)	10 (0.53%)
Fast-track THA (*n* = 239)			Fisher's Exact Test^d^, *p* = 0.319, phi = 0.086
Cemented (*n* = 72)	72 (100%)	0 (0%)
Uncemented (*n* = 167)	163 (97.6%)	4 (2.4%)

Note. THA = total hip arthroplasty. While some of the comparisons are statistically significant, the effect sizes are quite small.

^a^Body mass index was not routinely recorded prior to January 2007, and ASA classification and surgery duration were not routinely recorded prior to April 2004, and thus reduced sample sizes were available for these variables. ASA scores (3) and (4) were combined for analysis.

^b^Two outliers were excluded from the length of stay analysis, one patient was hospitalized 63 days with an infection developing on day 12 and the other was hospitalized for 95 days without infection.

^c^Because prosthesis type was confounded with surgery type (uncemented prostheses were more common among fast-track surgeries than among standard surgeries), the analysis of prosthesis type was conducted separately for each surgery type.

^d^Fisher's Exact Test was used due to the low expected cell frequencies in this analysis.

**Table 3:** Regression analysis of risk for infection-related revision surgery (*n* = 4,403).

Characteristic	OR	95% CI	*p* value
Age above 70 years	1.710	0.761, 3.838	0.194
Male gender	2.899	1.311, 6.410	0.009
Fast-track surgery	3.315	1.125, 9.772	0.030

Omnibus test of model coefficients: chi-square = 11.5, *p* = 0.009; Hosmer and Lemeshow test of goodness of fit: chi-square = 0.421, *p* = 0.810.
